# Evaluating HIV prevention efforts using semiparametric regression models: results from a large cohort of women participating in an HIV prevention trial from KwaZulu-Natal, South Africa

**DOI:** 10.7448/IAS.16.1.18589

**Published:** 2013-11-25

**Authors:** Handan Wand, Gita Ramjee

**Affiliations:** 1 The Kirby Institute, University of New South Wales, Sydney, New South Wales, Australia; 2 HIV Prevention Research Unit, Medical Research Council, Durban, South Africa

**Keywords:** HIV, women, South Africa, semiparametric regression

## Abstract

**Objective:**

To describe and quantify the differences in risk behaviours, HIV prevalence and incidence rates by birth cohorts among a group of women in Durban, South Africa.

**Methods:**

Cross-sectional and prospective cohort analyses were conducted for women who consented to be screened and enrolled in an HIV prevention trial. Demographic and sexual behaviours were described by five-year birth cohorts. Semiparametric regression models were used to investigate the bivariate associations between these factors and the birth cohorts. HIV seroconversion rates were also estimated by birth cohorts.

**Results:**

The prevalence of HIV-1 infection at the screening visit was lowest (20.0%) among the oldest (born before 1960) cohorts, while the highest prevalence was observed among those born between 1975 and 79. Level of education increased across the birth cohorts while the median age at first sexual experience declined among those born after 1975 compared to those born before 1975. Only 33.03% of the oldest group reported ever using a condom while engaging in vaginal sex compared to 73.68% in the youngest group; however, HIV and other sexually transmitted infection (STI) incidence rates were significantly higher among younger women compared to older women.

**Conclusions:**

These findings clearly suggest that demographic and sexual risk behaviours are differentially related to the birth cohorts. Significantly high HIV and STI incidence rates were observed among the younger group. Although the level of education increased, early age at sexual debut was more common among the younger group. The continuing increase in HIV and STI incidence rates among the later cohorts suggests that the future trajectory of the epidemic will be dependent on the infection patterns in younger birth cohorts.

## Introduction

The HIV epidemic continues to have a devastating effect in South Africa where the primary route of transmission still remains unprotected heterosexual contact [1]. Although the number of people living with the infection has increased dramatically since the early 1990s [2], intensification of HIV-related prevention activities only started in 2000 with expansion of the Voluntary Counselling and Testing campaign [3]. The widespread public availability of antiretroviral therapy was achieved in South Africa only after 2003 [4].

Women born before 1965 were at childbearing age and sexually active at the beginning of the epidemic, a time when there were no effective campaigns against HIV/AIDS in South Africa [4], whereas women born after 1965 were less than 25 years old and likely to be at lower risk. However, this younger group eventually contributed to rising HIV infection rates after 1993 [4]. Although there were few active HIV/AIDS campaigns when they were at their most reproductive age, we hypothesized that older women might have had longer exposure to the HIV/AIDS campaigns, while younger women, particularly those born after 1980, potentially had shorter exposure but may have been exposed to more effective HIV/AIDS prevention campaigns compared to those born before 1965. Therefore, characteristics and sexual behaviours may be different across birth cohorts.

Estimates of current sexual risk behaviours are important for assessing the impact of public health prevention strategies and may need to be regularly updated in order to track the changing profile of the epidemic. Monitoring new HIV infections among various subgroups such as age and/or birth cohorts, may provide important information on the spread of the infections. This crucial information may eventually contribute to a better understanding of the HIV epidemic at the national level. Estimates based on age at infection and birth cohorts complement each other. It is useful to monitor age at infection if the overall age is changing over time, while results from birth cohorts are important for understanding important features in HIV infection trends. Together they provide detailed insights into the epidemic so that the effectiveness of past/current HIV prevention programs can be assessed. It is crucial to reduce high risk sexual behaviours and target those practicing such high risk behaviours.

We first described changes in the key characteristics of the women who consented to be screened for a biomedical intervention trial across birth cohorts. The relationship of these characteristics and the birth cohorts were modelled using semiparametric regression models [5]. HIV seroconversion rates were also estimated by birth cohorts by adjusting for high risk sexual behaviours known to be associated with HIV acquisition.

## Methods

### Study population

The Methods for Improving Reproductive Health in Africa (MIRA) study was a randomized controlled open-label study comparing the effectiveness of the latex diaphragm plus lubricant gel with the provision of condoms alone for the prevention of heterosexual HIV acquisition among women (2003 to 2006). The methodology of the MIRA study has been described previously [6].

In Durban, the MIRA study was conducted at two clinics: in Umkomaas (on the south coast of KwaZulu-Natal province) and in Botha's Hill (30 km inland from Durban). The main eligibility criteria were: being sexually active; aged 18–49 years; being HIV negative (at enrolment); and being *Chlamydia trachomatis* and *Neisseria gonorrhoeae* negative at the screening (or willing to be treated if positive). All participants provided written informed consent at the screening visit.

### Study procedures

At screening, an interviewer-administered questionnaire covering demographics and sexual behaviour was completed. Participants received HIV pre-test counselling before being tested for HIV and sexually transmitted infections (STIs) at the screening visit. HIV diagnostic testing was carried out using two rapid tests (Determine HIV-1/2 (Abbott Laboratories, Tokyo, Japan) and Oraquick (Orasure Technologies, Bethlehem, PA, USA)) on whole blood from either finger-prick or venipuncture. Those found to be HIV infected were referred to appropriate referral clinics for HIV care. A urine specimen was collected for testing for *N. gonorrhoeae* and *C. trachomati*s by polymerase chain reaction (PCR) (Roche Pharmaceuticals, Branchburg, NJ, USA).

Participants underwent a pelvic examination, provided a blood sample for testing for syphilis (rapid plasma reagin (RPR) and *Treponema pallidum* haemagglutinin (TPHA), Randox Laboratories, Crumlin, UK) and herpes simplex virus 2 (HSV-2) (ELISA, FOCUS Diagnostics, Cypress, CA, USA), and provided a urine sample for pregnancy testing. After enrolment, at each quarterly follow-up visit, HIV and STI testing was carried out and curable STIs (if applicable) were treated.

The MIRA protocol was approved by the University of California at San Francisco Institutional Review Board Committee on Human Research. The study also received ethics approval from the Biomedical Research Ethics Committee of the University of KwaZulu-Natal, as well as the ethics review committees in all other local institutions and collaborating organizations. This study is registered with ClinicalTrals.gov, number NCT00121459.

Demographic characteristics, including age (years), level of education (years), age at first sex (years), marital status (legally married or not) and cohabitation status (whether the participant was living with her sexual partner or not), number of lifetime sexual partners, condom use (ever), contraception use and number of parities, were all determined at the screening.

### Statistical analyses

Birth cohorts were grouped in seven five-year groups: born before 1960, 1960–64, 1965–69, 1970–74, 1975–79, 1980–84 and on/after 1985. Trends across the groups were described using the median (inter-quartile range (IQR)) for continuous variables and percentages for categorical variables, but no formal statistical tests were performed.

Semiparametric regression models are modern statistical techniques that allow the observed data determine the functional form of the association between predictors and responses [5]. In this study, we used these flexible methods to investigate potential non-linear associations between the prevalence of HIV infection and birth cohorts. Other factors such as age at sexual debut (years), number of sexual partners and level of education (years) were also considered and analyzed. Significant non-linear effects were determined using visual inspection as well as the estimated effective number of degrees of freedom (i.e. the total influence of all observations). Higher degrees of freedoms (>2) were interpreted as evidence that a semiparametric regression model fit the data better than a fully parametric regression model such as logistic regression.

For example, year of birth (continuous covariate) is fitted to predict the risk of HIV seropositivity (binary outcome) using semiparametric logistic regression

$$\text{logit}[p(\text{HIV seropositive}|\text{birth cohorts})]=f{\left(\text{birth cohorts}\right)}_{i}+\varepsilon_{i}$$

where *f* is a real valued function. Based on this methodology, an image plot of the HIV cases was created as a smoothed function of birth cohorts. Similar models were used to assess the potential functional associations between the birth cohorts and other variables such as age at sexual debut, number of lifetime sexual partners, number of births and years of education.

Kaplan-Meier survival analyses were carried out to estimate the proportion of women progressing to HIV infection during the study (i.e. time of infection) and stratified by the tertiles of the birth cohorts (<1970, 1970–79, 1980+). Crude incidence rates were formally compared using the log-rank test. Time to seroconversion was defined as the difference between the calculated seroconversion date and the enrolment date. Women who were negative during the study (or discontinued/were lost to follow-up) were censored at the date of their last HIV test. As Padian *et al*. [6] state:

For participants who seroconverted, the time of seroconversion was defined as the time of first positive HIV test result. For cases in which one or more visits were missed in the intervals between the last negative and first positive tests, the time of seroconversion was assumed to be the visit containing the midpoint between these two time points.

Cox proportional hazard models were used to evaluate the influence of the birth cohorts on progression to HIV seroconversion and incidence of STIs, while simultaneously adjusting for other risk factors known to be associated with HIV and STIs [7]:

$$h_{i}(t)=\exp(\alpha +\beta_{i}(\text{birth cohorts}))$$

where *i* is a subscript for the *i* ^th^ observation for birth cohorts and *α* is the log-baseline hazard function.

Analyses were conducted using STATA 10.0 (College Station, TX, USA). Semiparametric smoothing models described in this section were fitted using the function *semipar ()* in the statistical software system *R* 2.15.1.

## Results

### Patterns of demographic, sexual and biological risk factors by birth cohorts

A total of 3492 women who consented to be screened for the MIRA trial were included in the analyses. Demographic, sexual and biological factors are given by birth cohort in Table 1. The prevalence of HIV infection at the screening visit was lowest among women born before 1960 (20%), steadily increased thereafter, peaked among those born in 1975–79 (approximately 53%), and declined again among those born in 1980–84 (approximately 42%) and on/after 1985 (approximately 22%). The prevalence of STIs (*C. trachomatis*, *N. gonorrhoeae*, syphilis) was lowest (approximately 6%) among those born before 1960 (oldest group). However, STI prevalence increased steadily and was highest (approximately 21%) among those born on/after 1985 (youngest group), while the majority of the older women tested positive for HSV-2 (89.00%, 81.89%, 81.62%, 81.40%, 76.75%, 66.22% and 43.34% for the seven birth cohorts, respectively). The median age at sexual debut tended to decrease across the birth cohorts from 18 (IQR 16–19) years among those born before 1960, to 16 (IQR 15–17) years in the youngest cohort born on/after 1985. There were substantial differences in patterns of marital and cohabitation status across the birth cohorts. Among the oldest women, 53.64% reported being married, compared to 7.01%, 2.88% and 0.93% of the three youngest birth cohorts, respectively; 69.09% of those born before 1960 were living with their sexual partners compared to 25.06%, 13.33% and 8.02% in the three youngest cohorts, respectively. Completing high school education rose from 0% in the oldest women to 33.9%, 34.61% and 25.00% in the three youngest birth cohorts, respectively. The likelihood of using any contraceptive methods increased with later birth cohorts (55.05%, 70.47%, 74.75%, 77.46%, 78.05%, 81.67% and 80.50% for the seven birth cohorts, respectively). Consistent with this result, the use of male condoms as the current contraceptive method was lowest in the earlier cohorts compared to the more recently born groups. Only 34.86% of those born before 1960 reported ever using a condom with vaginal sex compared with over 70% in the three youngest cohorts. Not surprisingly, the number of sexual partners and parity were higher among the older women compared with the younger women.

**Table 1:** Characteristics of women by birth cohort

	<1960 N = 110	1960–64 N = 257	1965–69 N = 409	1970–74 N = 559	1975–79 N = 770	1980–84 N = 1043	1985+ N = 324
HIV seropositive, n (%)	22 (20.00)	67 (26.07)	143 (34.96)	276 (49.37)	408 (52.99)	442 (42.38)	73 (22.53)
STI^1^, n (%)	7 (6.36)	25 (9.73)	48 (11.74)	66 (11.81)	124 (16.10)	212 (20.33)	68 (20.99)
HSV-2 at screening, n (%)	97 (89.00)	208 (81.89)	333 (81.62)	455 (81.40)	591 (76.75)	690 (66.22)	140 (43.34)
Language spoken at home, n (%)							
Zulu	102 (92.73)	241 (93.77)	369 (90.22)	522 (93.38)	735 (95.45)	998 (95.69)	314 (96.91)
Other	8 (7.27)	16 (6.23)	40 (9.78)	37 (6.62)	35 (4.55)	45 (4.31)	10 (2.09)
Education, n (%)							
High school and above	–	14 (5.45)	65 (15.89)	122 (21.82)	261 (33.90)	361 (34.61)	81 (25.00)
Less than high school	110 (100.00)	243 (94.55)	344 (84.11)	437 (78.18)	509 (66.10)	682 (65.39)	243 (75.00)
Age at first sex							
16 years or younger	42 (38.18)	99 (38.52)	172 (42.05)	201 (35.96)	277 (35.97)	417 (39.98)	191 (58.95)
Cohabitation status							
Legally married	59 (53.64)	129 (50.19)	139 (33.99)	114 (20.39)	54 (7.01)	30 (2.88)	3 (0.93)
Living with a regular sexual partner	76 (69.09)	162 (63.04)	233 (56.97)	241 (43.11)	193 (25.06)	139 (13.33)	26 (8.02)
Lifetime sexual partner							
1	26 (23.64)	59 (22.96)	70 (17.11)	116 (20.75)	151 (19.61)	296 (28.38)	139 (42.90)
2	31 (28.18)	61 (23.74)	118 (28.85)	141 (25.22)	225 (29.22)	306 (29.34)	96 (29.63)
3	23 (20.91)	56 (21.79)	89 (21.76)	133 (23.79)	167 (21.69)	213 (20.42)	50 (15.43)
4+	30 (27.27)	81 (31.52)	132 (32.27)	169 (30.23)	227 (29.48)	228 (21.86)	39 (12.04)
Contraception currently used							
None	49 (44.95)	75 (29.53)	103 (25.25)	126 (22.54)	169 (21.95)	191 (18.33)	63 (19.50)
Any contraception	60 (55.05)	179 (70.47)	305 (74.75)	433 (77.46)	601 (78.05)	851 (81.67)	260 (80.50)
Male condom	21 (19.27)	69 (27.17)	146 (35.78)	224 (40.07)	373 (48.44)	566 (54.32)	171 (52.94)
Long term (injectables)	9 (8.26)	37 (14.57)	124 (30.39)	230 (41.14)	336 (43.64)	433 (41.55)	117 (36.22)
Ever used male condom							
As contraceptive	36 (33.03)	125 (49.21)	249 (61.03)	372 (66.55)	589 (76.49)	825 (79.17)	239 (73.99)
With vaginal sex	38 (34.86)	126 (49.61)	254 (62.25)	377 (67.44)	598 (77.66)	838 (80.42)	238 (73.68)
Parity							
0	1 (0.92)	8 (3.15)	13 (3.19)	20 (3.58)	91 (11.82)	246 (23.61)	130 (40.25)
1	8 (7.34)	19 (7.48)	57 (13.97)	143 (25.58)	341 (44.29)	626 (60.08)	181 (56.04)
2	13 (11.93)	31 (12.20)	86 (21.08)	181 (32.38)	241 (31.30)	151 (14.49)	12 (3.72)
3	24 (22.02)	51 (20.08)	110 (26.96)	128 (22.90)	72 (9.35)	18 (1.73)	–
4+	63 (57.80)	145 (57.09)	142 (34.80)	87 (15.56)	25 (3.25)	1 (0.10)	–

1 *Chlamydia*, gonorrhoea, syphilis; HSV-2, herpes simplex virus 2; STI, sexually transmitted infection.

### Semiparametric logistic regression models for HIV seroprevalence and birth cohorts

**Figure 1:**
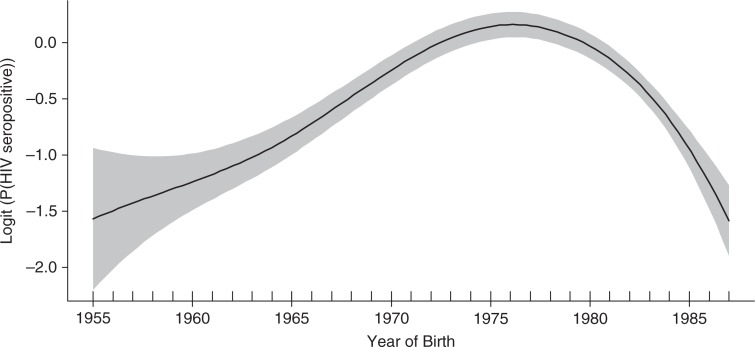
Non-linear association between birth years and HIV seropositivity.

**Figure 2:**
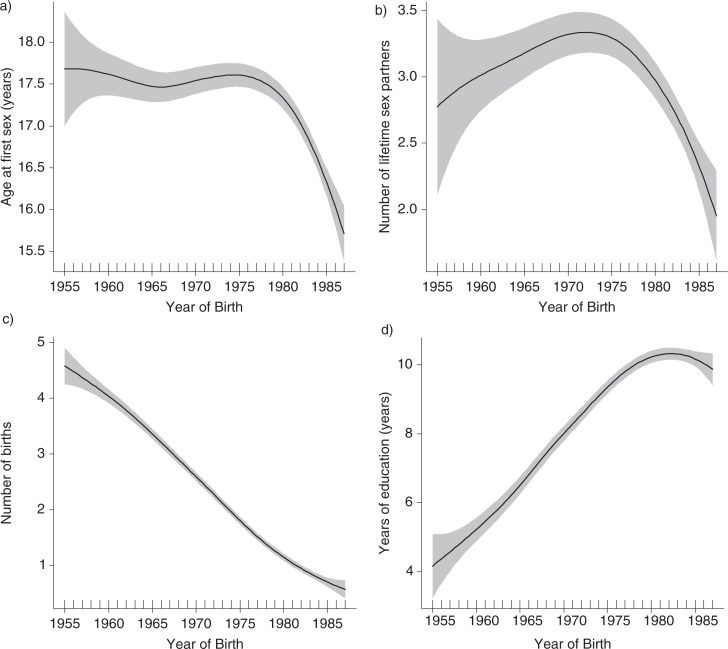
Association between birth years and age at first sex (a), number of lifetime sexual partners (b), parity (c) and years of education (d).

Figure 1 shows the functional relationship between the year of birth and log of the odds ratios of HIV prevalence. Inverted U-shaped associations were observed between the year of birth and HIV seropositivity with an estimated 4.0 degrees of freedom, supporting a non-linear relationship. In Figure 2 we show the fits to birth cohorts and age at first sex (years), number of lifetime sexual partners, number of births and level of education (years). Age at sexual debut remained largely unchanged among those born before 1975, then sharply decreased, reaching the lowest age in the youngest cohort (Figure 2a). Trends in the number of sexual partners also showed a non-linear association across the birth cohorts, with the highest rates among those born in 1970–75 (Figure 2b). A strong linear negative association was observed between the number of births and year of birth (Figure 2c), while level of education steadily increased across the birth cohorts, reaching the highest level among those born after 1980 (Figure 2d).

### Incidence of HIV, STIs by birth cohorts

Table 2 summarizes the crude incidence rate ratios and results from the unadjusted and adjusted Cox proportional hazard analyses by tertiles of the birth cohorts. A total of 1485 women enrolled in the MIRA trial from the Durban sites and 148 (10.0%) HIV infections occurred during the study. The rate of HIV acquisition was higher among younger women compared to older women (9.6 per 100 person-years, 6.6 per 100 person-years and 3.3 per 100 person-years, respectively, in the highest to lowest tertiles of the birth cohorts, *p*<0.001).

**Table 2:** HIV and STI incidence^0^ by birth cohort

	Crude incidence rate per 100 PY (95% CI)	*p*-Value^1^	Unadjusted HR (95% CI)	*p*-Value^2^	Adjusted^3^ HR (95% CI)	*p*-Value^2^
	**HIV incidence**					
Birth cohort		<0.001				
Born before 1970	3.34 (2.15, 5.17)		1		1	
Born 1970–80	6.65 (5.00, 8.82)		2.03 (1.21, 3.42)	0.008	1.82 (1.06, 3.11)	0.029
Born after 1980	9.60 (7.71, 12.00)		2.88 (1.77, 4.71)	<0.001	2.27 (1.33, 3.87)	0.003
	**STI incidence**					
Birth cohort		<0.001				
Born before 1970	13.27 (10.88, 16.20)		1		1	
Born 1970–80	13.70 (11.43, 16.42)		1.04 (0.79, 1.36)	0.799	0.99 (0.75, 1.31)	0.973
Born after 1980	24.00 (21.00, 27.35)		1.78 (1.40, 2.26)	<0.001	1.66 (1.26, 2.18)	<0.001

0 93% of women completed the scheduled closing visit, 5% were lost to follow-up and 1.7% withdrew

1 log-rank test

2 Cox proportional regression

3 adjusted for number of lifetime sex partners (<2, 2, 3, 4+), age at sexual debut (16 years of age or younger vs 17+), cohabitation status (cohabiting with a sexual partner vs not), level of education (less than high school vs at least high school) and ever used condom with vaginal sex (yes/no); CI, confidence interval; HR, hazard ratio; PY, person-years; STI, sexually transmitted infection.

We also used unadjusted and adjusted Cox proportional hazards modelling to examine the associations between the risk of HIV infection and the birth cohorts. This analysis was not an attempt to assess causal links for HIV seroconversion (or STI incidence) but rather to control statistically for confounding for demographic and sexual risk behaviour differences (Table 1). Women in more recent birth cohorts (born after 1970) had a significantly higher risk of HIV acquisition compared to those born before 1970 (hazard ratio (HR): 2.88, 95% confidence interval (CI): 1.77, 4.71 and HR: 2.03, 95% CI: 1.21, 3.42, respectively). This association was slightly attenuated when the analysis was adjusted for level of education, number of sexual partners, cohabitation status and condom used in last sexual act during the follow-up (HR: 2.27, CI: 1.33, 3.87 and HR: 1.82, 95% CI: 1.06, 3.11, respectively).

Younger age was also associated with increased risk of STI compared with older women. Rates of STI positivity increased among those who were in the earlier cohort compared to the later cohort (24.0 per 100 person-years vs approximately 13.0 per 100 person-years, respectively, *p*<0.001). Those born after 1980 were particularly significantly associated with increased risk of STIs compared with the later cohorts (HR: 1.66, 95% CI: 1.26, 2.18 and HR: 0.99, 95% CI: 0.75, 1.31, respectively).

## Discussion

After more than 30 years of research on risk factors, we now have a good understanding of how HIV is acquired. In order to demonstrate the impact of HIV prevention messages on subsequent acquisition of HIV and other STIs, it is necessary to show that prevention messages bring about changes in sexual behaviours.

The current study has focused on describing and quantifying the associations between demographics, sexual behaviours, HIV seroprevalence and incidence rates by birth cohorts rather than by age. However, one can roughly translate the birth cohorts and calendar year of HIV infection into the age of each individual. For example, people born in 1960–64 and infected with HIV in 2003, correspond to the age interval for “39–43 years”. In addition, because age is strongly correlated with the year of birth (Spearman's correlation coefficient −0.99, *p*<0.001), in the presence of the “birth cohorts”, age was not determined to be the independent predictor for incidence of HIV infection.

Our study provided quantitative assessments of the HIV epidemic among a select group of women in KwaZulu-Natal province in South Africa by birth cohorts, thus providing a sound basis for informing targeted public health policy. We detected clear differences between generations in terms of risk of HIV infection, condom use and age at sexual debut among other metrics, in agreement with previously published South African data which found that age at first sex was earlier among later birth cohorts, and that condom use increased among later-born study participants [8].

It is clear that the younger age groups are experiencing delays in the onset of HIV infections (until they are sufficiently sexually active). The prevalence of HIV seropositivity at the screening visit was low and similar between the oldest (born before 1960) and the youngest (born after 1985) birth cohorts, being 20% and 22.53%, respectively; however, time to HIV seroconversion or acquisition of other STIs during the study was much shorter in younger groups compared to older groups (9.60 per 100 person-years and 3.34 per 100 person-years, respectively).

Encouragingly, level of education increased across the birth cohorts; for example, in the most recent birth cohorts (i.e. those born since 1975), the level of education and the rates of condom use were much higher compared to those in earlier cohorts. The increase in rates of condom use among later birth cohorts is in accordance with nationally representative data on this metric [9]. Increased levels of education may be associated with a decline in the risk of acquiring HIV [8,10]. However, this younger cohort also had a lower age at first sex compared to those born earlier. Such a trend may paradoxically result in an increase in HIV incidence over time among younger women relative to older women. The high incidence of non-HIV STIs among the youngest cohort is of particular concern, given the hypothesis that STIs may increase vulnerability to HIV [11].

Provision of appropriate sex, HIV and general education prior to the onset of sexual activity may be effective in preventing transmission of HIV by increasing age at first sex [12,13], and by increasing condom use and reducing the number of sexual partners for those who are sexually active [14]. Intensive HIV prevention campaigns on these key risk factors are thought to have contributed to the reduction in HIV prevalence in Uganda [15]. In the South African context, Pettifor *et al*. [16] noted that participation in the “Lovelife” youth HIV prevention program was associated with a lowered risk of HIV infection among youth in a nationally representative household survey.

Understanding the associations between sexual behaviours and the transmission of HIV and STIs is the primary focus of this research and may provide greater insight into the dynamics of the epidemic. However, the current challenge is to interpret these results in terms of the effectiveness of the prevention efforts to prioritize prevention strategies. Our study investigated the changes in risk behaviours of women by birth cohort to assess the impact of interventions using the birth cohorts as a surrogate for period effect in relation to risk for HIV infection.

Investigation of the impact of birth cohorts on the HIV epidemic is usually performed using standard statistical techniques [17–19]. To our knowledge, this is one of the first studies to investigate the impact of birth cohorts on HIV seropositivity using advanced smoothing techniques.

Our study has several limitations and therefore the results should be interpreted with caution. First of all, the current analysis was not an attempt to quantify causal links for HIV seroconversion but rather to control statistically for confounding for demographic and sexual risk behaviour differences based on the literature [7,20–23]. Potential confounders were determined and included in adjusted Cox regression models. Our study population consisted of a group of women enrolled in an HIV prevention trial, who might therefore be at higher risk for HIV infection compared to the general population. Data on several potentially important risk factors which could influence some of the metrics analyzed here were not collected (such as age at menarche, which can be associated with timing of sexual debut [7]) during the MIRA study. We also cannot rule out the effects of unmeasured characteristics such as poverty, cultural differences, multiple or concurrent sex partners and commercial sex, on our findings. No data concerning migration, socio-economic status, sexual behaviour or HIV status of male partners were collected in this study.

We must continue to monitor trends in HIV prevalence and incidence in this region, and combine this with active monitoring of the sexual behaviours reported in the same population. This is a need to ensure that consistent and continuous HIV prevention messages are delivered, in line with the social and cultural context of the community where research is conducted.
